# From the latin “re-cordis, passing through the heart”: autonomic modulation differentiates migraineurs from controls when recounting a significant life event

**DOI:** 10.1007/s10072-024-07739-7

**Published:** 2024-08-27

**Authors:** Sara Guidotti, Paola Torelli, Giordano Ambiveri, Alice Fiduccia, Matteo Castaldo, Carlo Pruneti

**Affiliations:** 1 Clinical Psychology, Clinical Psychophysiology, and Clinical Neuropsychology Labs., Dept. of Medicine and Surgery, University of Parma, Parma, Italy; 2 Headache Center, Neurology Unit, University Hospital of Parma, Parma, Italy; 3 San Giacomo Private Hospital, Ponte dell’Olio, Piacenza, Italy; 4 Center for Sensory-Motor Interaction (SMI), Department of Health Science and Technology, School of Medicine, Aalborg University, Aalborg, Denmark

**Keywords:** Migraine, Psychophysiology, Autonomic imbalance, Heart rate variability, Stress response

## Abstract

**Objective:**

The literature on clinical psychophysiology highlights the possibility of using Heart Rate Variability (HRV) as an index of psychophysical balance and resilience to stress. This study investigates the differences in stress reactivity and subsequent recovery between a group of migraineurs and healthy controls.

**Methods:**

Socio-demographic (i.e., sex, age, profession, marital status, and level of education) and psychophysiological (HR and HRV) measures of a group of thirty subjects with migraine (26 migraineurs without aura (86.7%), 2 migraineurs with aura (6.7%), and 2 migraineurs with and without aura (6.7%)) and from thirty healthy control subjects were collected. In particular, HRV was analyzed through frequency-domain parameters, including Low-Frequency (LF; 0.04–0.15 Hz) and High-Frequency (HF; 0.15–0.4 Hz) bands as well as LF/HF ratio during a Psychophysiological Stress Profile (PSP) structured in seven phases: (1) Baseline, (2) Objective stressor 1 (Stroop Test), (3) Rest 1, (4) Objective stressor 2 (Mental Arithmetic Task), (5) Rest 2, (6) Subjective stressor (recount a significant life event), and (7) Rest 3. The LF, HF, and LF/HF ratio values were transformed into a logarithmic scale (i.e., log-LF, log-HF, and log LF/HF ratio). Additionally, LF and HF were converted into normalized units (0-100) (i.e., LF% and HF%) which, in turn, were used to obtain reactivity and recovery to stress through delta values (Δ) calculation.

**Results:**

Subjects with migraine reported greater ΔLF% levels of reactivity and recovery to subjective stressor, demonstrating a prevalence of sympathetic activity while recounting a personal life event. At the same time, a lowering of the same values was found in the subjects of the group control.

**Discussion:**

Our results underline the importance of conducting a psychophysiological assessment in patients with headaches because reduced stress management skills could influence the clinical manifestations of the disease, considering stress as one of the most common triggers for migraine patients.

**Supplementary Information:**

The online version contains supplementary material available at 10.1007/s10072-024-07739-7.

## Introduction

Migraine is a complex multifactorial neurological disorder that represents one of the most disabling conditions [1]. Despite the recent improvement in knowledge about migraines and the development of new preventive pharmacological options, the burden of migraines has continued to enlarge in recent years [2]. In 2017, migraine became the leading cause of years lived with disability in people aged 15 to 49, with a prevalence of approximately 15% of the general population [3].

As migraine impacts how the central nervous system (CNS) processes sensory information, it can lead to various symptoms, including sensitivity to light and sound, nausea, dizziness, and aversion to certain smells [4]. The disorder is marked by distinct phases, including preictal, ictal, postictal, and interictal. Each phase is linked to the activation of specific regions of the brain and is associated with a variety of clinical manifestations [4].

The pathophysiology of migraine is currently not yet fully understood [5]. Nonetheless, the most accredited hypothesis contemplates the involvement of the trigeminovascular pathway to be primary [6]. The path formed by the trigeminal nerve, with its axonal projections to the meninges and blood vessels, transmits nociceptive information to different regions of the brain, including the cortex [7]. Another hypothesis that received scientific validation concerns central sensitization, a mechanism widely studied in migraine pathophysiology [8–10]. Referring to research, migraine is characterized by elevated reactivity of CNS neurons to normal or subthreshold afferent inputs [11]. In general, sensitization is a common feature of chronic pain conditions as it triggers CNS excitability with repeated nociceptive input, resulting in widespread hyperalgesia and/or allodynia, as well as alterations in cognitive and emotional-affective processing [12, 13].

In addition to eating poorly on time, tiredness, and lack of sleep, the presence of stress/tension is among the most common precipitating factors the scientific community recognizes [14–16]. The stress response is a physiological and hormonal reaction that prepares the body to cope with stress by activating its energy resources [17]. Specifically, the sympathetic branch of the autonomic nervous system (ANS) induces the release of neurotransmitters, such as adrenaline and norepinephrine which, in turn, favor the mobilization of glucose, and the increase in heart rate (HR) and blood pressure [18]. At the same time, the activation of the hypothalamic-pituitary-adrenal (HPA) axis favors the release of stress hormones (i.e., cortisol) which further enhance physical and cognitive performance [19, 20]. Nonetheless, prolonged stress can negatively affect an individual’s health, such as compromising the immune and cardiovascular systems. Several studies highlighted that stress is higher among migraine patients than healthy patients and above the clinical threshold level [21, 22]. The impact of stress on migraine patients may be higher, as they show a lower adaptability to changes (i.e., sleep changes, lifestyle changes, hormonal changes, dietary changes, etc.). Indeed migraine is characterized by an augmented cortical excitability [23]. On top of this, stress was confirmed as a primary factor in triggering and perpetuating migraine attacks [24–26] in nearly 70% of migraine patients [27], as demonstrated to contribute to the severity and frequency of migraine attacks [14, 15, 26]. In this regard, the predominant role of autonomic imbalance was manifested [16, 26, 28, 29]. Therefore, migraine has a great disability and burden, which can be a stressor itself, creating a vicious circle between migraine attacks and stressful events [15].

Recent studies found HR variability (HRV) to be a useful parameter for the evaluation of autonomic imbalance connected to the chronic stress response [30–32]. HRV is the variation in time between consecutive heartbeats (RR intervals) [33]. It consists of coupling and synchronizing the cardiac rhythm with the phases of respiration. Deep, regular breathing was validated to augment HR fluctuation and respiratory sinus arrhythmia, and it appears capable of optimizing the balance between the sympathetic (SNS) and parasympathetic (PNS) nervous systems [34]. The two components of the autonomic nervous system (SNS and PNS) are also known as the fight-or-flight mechanism and the tend-and-befriend behaviors, respectively [35, 36]. In other words, a higher prevalence of SNS activity reflects fight-or-flight behaviors, while PNS activity is associated with tend-and-befriend behaviors as well as relaxation [37]. HRV analysis is commonly conducted in either the time domain or frequency domain [38, 39]. In frequency domain analysis, the HR oscillation is divided into four bands: (1) ultra-low frequencies (ULF), (2) very low frequencies (VLF), (3) low frequencies (LF), and (4) high frequencies (HF). LF power (0.04–0.15 Hz) can be produced by both the PNS and SNS, as well as blood pressure regulation via baroreceptors, primarily by the PNS, or by baroreflex activity alone. On the other hand, HF power (0.15–0.4 Hz) is equivalent to the well-known respiratory sinus arrhythmia and is considered to represent vagal control of HR [40] and primarily reflects the PNS activity [35]. In addition, some researchers attested that the LF/HF ratio reflects both sympathovagal balance and sympathetic modulations [41], being sensitive to mental stress [42]. The power of the LF and HF spectral components of HRV can be expressed as absolute (ms^2^) or normalized units (0-100). The normalization procedure has proved crucial to the interpretation of data [43].

Research suggests that an optimal level of HRV is associated with self-regulatory capacity, adaptability, and resilience, whereas decreased vagal tone is a risk factor for cardiovascular disease and all-cause mortality [38, 44, 45]. Studies further linked decreased vagal tone to increased inflammation and less ability to control pain, especially in patients with headaches [46, 47]. A systematic review with meta-analysis attested that patients with migraines manifested a decrease in both sympathetic and parasympathetic modulation, observed by enlarged P-wave dispersion, longer QTc intervals, and a decrease in the rate of deep breathing, with no significant differences in the time-domain analyses of HRV [48]. Moreover, during the ictal period, episodic migraine patients presented a decrease in parasympathetic modulation documented by lower SDNN (a time-domain parameter) and in sympathetic modulation as reported by LF [49, 50].

HRV analysis was recommended for both long-term (24 h) and short-term (5 min) studies [30]. Although 24-hour HRV analysis is useful for increasing frequency resolution [43], its application in samples of volunteer subjects is difficult. Particularly, changes in the physical or mental states, environments, and even noises in ambulatory recordings can severely affect the results of HRV analysis [41]. In contrast, rigorous experimental control is possible for short recordings (i.e., a few minutes) carried out in well-controlled environments [41].

Long-term recordings are carried out with dynamic electrocardiogram (ECG) according to Holter [51], while short-term recordings can be made with precordial ECG [41]. Some recording devices do not use the traditional QRS complex of an ECG to calculate HR and HRV. To illustrate, PPG uses a photoelectric sensor that estimates changes in blood volume to calculate HR. The PPG technique involves using a photocell (such as an infrared light-emitting diode) positioned on an easily accessible tissue area with blood capillaries (i.e., finger or earlobe). The energy emitted by an infrared source passes through the tissue and is reflected on it. The amount of light can then assess changes in blood volume (due to heartbeats) in an area reflected to the photodetector, and, thus, form the basis for estimating heartbeats [30, 52].

Several studies corroborated PPG as an accurate method for beat-to-beat HR information in different patient groups [53]. Except for breathing protocols based on the resonant frequency exercises which documented lower accuracy of PPG compared to ECG [43], a reasonable correlation between these two measures was validated [53]. Specifically, a study by Parak et al. [54], demonstrated that PPG had an average beat-to-beat absolute error after applying an artifact correction algorithm of 5.94 ms, indicating similar accuracy to ECG and suggesting potential use in a clinical setting [53, 55]. Lastly, although these two measures may diverge under acute stress conditions [56], PPG proved to reliably assess acute stress reactivity [57]. Specifically considering the reactivity to stress, there is evidence that the calculation of the difference between induced stress and baseline as well as that between the rest phase and the previously administered stressor can manifest some psychophysiological phenomena that are not appreciated only in the baseline phase [58].

In light of this evidence, the objective of the present study was to analyze HRV in migraine patients recording a psychophysiological profile. In particular, a group of migraineurs may demonstrate differences in HRV values under various types of stressor stimuli compared to a group of controls.

## Materials and methods

### Participants and procedure

This case-control study enrolled people between the ages of 18 and 65. People who decided to voluntarily participate responded to posters allowing them to scan a QR code and access the Outlook Calendar to book an appointment.

The clinical group was composed of people diagnosed with migraine. The headache diagnosis was made by a board-certified neurologist using the International Headache Society (IHS) criteria [25]. Criteria for inclusion in the study were age > 18 years old, completion of informed consent, and no history of neuropsychiatric syndromes (i.e., previous head trauma, epilepsy, etc.) and/or physical diseases (i.e., sensory disturbances of sight and/or hearing) that may limit the protocol procedure. Exclusion criteria were the presence of pain in the musculoskeletal and dental systems, history of neck, temporomandibular joint, shoulder, or back surgery, the presence of other neurological deficits or a rheumatoid disorder, the assumption of analgesics within 24 h before the investigation of psychotropic drugs with rebound effects on the ANS in the last three months (i.e., tricyclic antidepressants; antipsychotics; norepinephrine–dopamine reuptake inhibitors, such as bupropion; serotonin modulators, such as mirtazapine and trazodone; serotonin-norepinephrine reuptake inhibitors, such as venlafaxine and duloxetine), and overuse headache medications as defined by the IHS.

The control group consisted of age-matched volunteers. Participants in the control group had no headaches for at least 3 months before the study and had experienced occasional mild headaches (< 5 times per year), for which they had never sought medical care. Criteria for inclusion in the study were age > 18 years old, completion of informed consent, and no history of neuropsychiatric syndromes (i.e., previous head trauma, epilepsy, etc.) and/or physical diseases (i.e., sensory disturbances of sight and/or hearing) that may limit the protocol procedure.

The researchers explained the purpose of the study as well as the psychophysiological assessment procedure, without specifying the single phases so as not to influence the stress response. Subsequently, participants were offered the option to book another appointment with a licensed clinical psychologist to receive an exhaustive commentary on the reports.

The Ethical Committee of the University of Parma approved the study (protocol number: 118310/2024). The research was conducted following the Guidelines for Good Research Practice of the University of Parma (2020). All procedures were conducted following the 1964 Declaration of Helsinki of the World Medical Association as well as the 2005 Universal Declaration on Bioethics and Human Rights defined by the UNESCO regarding research involving human participants.

### Measures

All enrolled people underwent a *Psychophysiological Stress Profile* [59]. The PSP consists of the execution of a 14-minute assessment including 7 phases (2 min each), after a 4-minute adaptation phase in which the subject is asked to sit quietly in a chair. During the experiment, patients were asked to remain as still as possible, with their feet placed on the ground at 45 degrees and their arms on the armrests. The room temperature is maintained between 19 and 21 °C for the entire duration of the recording. The seven phases include (1) Baseline, the person is asked to remain still and with eyes closed; (2) Objective stressor 1, a computerized version of the Stroop Test is proposed; (3) Rest 1, the person is asked to relax as much as possible; (4) Objective stressor 2, the Mental Arithmetic Task is proposed, which consists of mental calculations of serial subtraction (e.g., subtract consecutively the number 7 from 1008); (5) Rest 2, as described in Recovery 1; (6) Subjective stressor, the person is asked to briefly describe a significant life event; and (7) Rest 3, as described in Recovery 1.

Biograph Procomp Infiniti 5.0 (Thought Technology Ltd., Canada) was used to collect data regarding HR and Blood Volume Pressure (BVP) with a blood volume pulse sensing sensor (also known as PPG), using a cuff positioned on the finger of the right upper limb. PPG optically detects pulse waves by assessing changes in light absorption caused by blood flow [60]. Thus, the BVP sends infrared light to the skin surface and measures the amount of reflected light. The more blood there is in the skin, the more light is reflected.

The HRV indices analyzed in the frequency domain were low-frequency (LF, 0.04–0.15 Hz) and high-frequency (HF, 0.15–0.40 Hz) bands, obtained from the nonparametric method fast Fourier transform (FFT). The power spectrum was subsequently quantified into various frequency-domain measurements as defined previously (Table 1) [41]. Due to the skewed distribution of frequency domain variables, a natural logarithmic transformation was applied (log-LF and log-HF, respectively). Both measurements contribute to the formulation of the fraction between LF and HF (LF/HF ratio), which is also transformed into a natural logarithmic scale (log-LF/HF ratio). Additionally, LF and HF were normalized by the percentage of total power to detect SNS and PNS influence on HRV (LF% and HF%, respectively) [61]. Specifically, normalized values for HRV were obtained by calculating the percentage of LF and HF power relative to the total spectrum power minus the VLF band (< 0.04 Hz). The normalization procedure was performed to minimize total power variations in the absolute values of LF and HF [61].

**Table 1:** Description of HRV values

Variable	Units	Definition	Frequency Range
VLF	ms^2^	Power in VLF range	0.003–0.04 Hz
LF	ms^2^	Power in LF range	0.04–0.15 Hz
HF	ms^2^	Power in HF range	0.15–0.4 Hz
LF/HF	ratio	LF (ms^2^)/HF (ms^2^)	
LF%	nu	LF power in normalized units: LF/(total power - VLF) X 100	
HF%	nu	HF power in normalized units: HF/(total power - VLF) X 100	

Legend: VLF = Very-Low Frequency; LF = Low Frequency; HF = High Frequency; nu = Normalized units

### Statistical analysis

All statistical analyses were performed using SPSS (Version 28.0.1.0; IBM Corp, Armonk, NY, USA). First, differences in socio-demographic variables (i.e., gender, age, marital status, educational level, and occupation) between patients and control groups were assessed at baseline, using a Chi-Squared Test or an Independent Samples T-Test.

Concerning the psychophysiological values, all the assumptions for the conduction of parametric statistics were respected for HR and HRV (i.e., HR, log-LF, log-HF, log-LF/HF ratio, LF%, and HF%). A repeated-measures ANOVA was conducted to compare HR, log-LF, log-HF, log-LF/HF ratio, LF%, and HF% between groups in every phase of the PSP (i.e., Baseline, Objective stressor 1, Rest 1, Objective stressor 2, Rest 2, Subjective stressor, and Rest 3), one at time. Post-hoc analyses were made using the Bonferroni method.

Furthermore, delta (Δ) values ​​were calculated for LF% and HF% to have psychophysiological reactivity and recovery according to the recommendations of Laborde and colleagues [40]. Reactivity was obtained by calculating the difference between the stress phases and baseline (i.e., Objective Stressor 1 – Baseline = Reactivity 1; Objective Stressor 2 – Baseline = Reactivity 2; and Subjective Stressor – Baseline = Reactivity 3) to quantify changes in participants’ mean LF and HF HRV percentage values ​​(LF% and HF%, respectively) during stress induction (120-s) compared to the participant’s baseline values ​​(120-s). Similarly, reactivity was obtained by calculating the difference between the rest phase and stress induction (i.e., Recovery 1 - Objective Stressor 1 = Recovery 1; Recovery 2 - Objective Stressor 2 = Recovery 2; and Recovery 3 - Subjective Stressor = Recovery 3) to quantify the changes in the participants’ mean LF and HF percent HRV values ​​during the recovery phase (120-s) compared to the participant’s value during the stress induction (120-s).

Consequently, an Independent Samples T-Test was adopted to compare the delta values (i.e., Reactivity 1, Reactivity 2, Reactivity 3, Recovery 1, Recovery 2, and Recovery 3) between control and migraine groups.

## Results

### Sample characteristics and comparison between patients and controls

The patient group was composed of 26 patients with migraine without aura (86.7%), 2 patients with migraine with aura (6.7%), and 2 patients with migraine with and without aura (6.7%).

Table 2 shows the comparison between groups. No significant differences emerged in the socio-demographic variables, except for gender which highlights a higher prevalence of women in the sample of migraineurs. Comparisons between migraineurs and controls in HR, log-LF, log-HF, log-LF/HF ratio, LF%, and HF% HRV scores are reported in Table 3. None of the analyses reached statistical significance, although LF% and HF% approached the threshold of 0.05.

**Table 2:** Comparisons of socio-demographic characteristics between the control and migraine group

Variable	Control group (n=30)	Migraine group (n=30)	T or X^2^	p
Age, *M (SD)*	28.40 (10.70)	29.07 (10.34)	t(59) = 0.25	0.34
Sex, *N* (%)			χ^2^ (1, *N* = 59) = 8.86	0.01
Male	16 (54.8%)	5 (16.1%)		
Female	14 (45.2%)	25 (83.9%)		
Marital status, *N* (%)			χ^2^ (1, *N* = 59) = 0.10	0.29
Single/Unmarried	24 (80.6%)	23 (77.4%)		
Married/Cohabitant	6 (19.4%)	7 (22.6%)		
Education Level, *N* (%)			χ^2^ (2, *N* = 59) = 1.35	0.65
Middle school	1 (3.2%)	3 (9.7%)		
High school	13 (41.9%)	14 (48.4%)		
University	16 (54.8%)	13 (41.9%)		
Current Occupation, *N* (%)			χ^2^ (1, *N* = 59) = 0.08	0.45
Student	20 (64.5%)	21 (67.7%)		
Employed	10 (35.5%)	9 (32.3%)		

**Table 3:** Comparisons of HRV values between the control and migraine group

	Control group (n = 30)							Migraine group (n = 30)							Effects	
	Baseline	Objective Stress 1	Rest 1	Objective Stress 2	Rest 2	Subjective Stress	Rest 3	Baseline	Objective Stress 1	Rest 1	Objective Stress 2	Rest 2	Subjective Stress	Rest 3	Time x group F	p
HR (bpm)	73.32 ± 12.53	85.96 ± 13.01	72.81 ± 10.82	86.31 ± 12.56	72.94 ± 10.53	90.14 ± 11.13	73.16 ± 10.95	72.88 ± 10.14	85.49 ± 12.83	72.67 ± 8.50	85.96 ± 13.85	71.81 ± 7.96	88.71 ± 11.98	73.14 ± 8.75	0.03	0.86
log-LF (ms^2^)	5.52 ± 1.11	5.37 ± 1.27	5.29 ± 1.23	5.84 ± 1.20	5.49 ± 1.27	6.75 ± 1.44	5.70 ± 1.07	4.87 ± 1.11	5.67 ± 1.23	5.20 ± 1.37	6.08 ± 1.25	5.12 ± 1.09	6.73 ± 0.87	5.59 ± 1.56	0.32	0.57
log-HF (ms^2^)	5.00 ± 1.10	4.92 ± 1.60	5.08 ± 1.17	5.33 ± 1.61	5.38 ± 0.93	6.84 ± 1.68	5.56 ± 1.09	4.85 ± 1.04	5.29 ± 1.48	5.31 ± 1.47	5.78 ± 1.83	5.02 ± 1.10	6.74 ± 1.52	5.50 ± 1.57	1.68	0.20
log-LF/HF ratio	0.41 ± 1.89	0.45 ± 0.81	0.24 ± 1.04	0.51 ± 0.83	0.11 ± 1.05	-0.09 ± 0.68	0.14 ± 0.70	0.02 ± 1.10	0.38 ± 0.71	0.04 ± 1.00	0.31 ± 0.95	0.10 ± 0.74	-0.01 ± 1.02	0.19 ± 1.14	2.71	0.11
LF% (nu)	46.20 ± 18.51	46.11 ± 16.27	41.58 ± 21.03	46.92 ± 15.60	41.44 ± 18.10	34.95 ± 11.86	41.92 ± 13.78	36.32 ± 18.42	40.64 ± 15.26	37.43 ± 19.18	43.56 ± 16.55	35.24 ± 13.25	38.77 ± 18.25	37.37 ± 15.47	3.43	0.06
HF% (nu)	32.09 ± 18.44	30.90 ± 3.55	35.28 ± 18.71	27.97 ± 12.89	36.44 ± 17.28	39.08 ± 12.51	37.74 ± 12.11	39.28 ± 18.91	31.78 ± 12.88	38.60 ± 17.32	32.98 ± 16.65	35.09 ± 15.60	38.88 ± 18.34	37.53 ± 14.49	3.01	0.08

*Note*: Data are presented as mean ± standard deviation. *Legend*: HR = Heart Rate; log-LF = Low-Frequency band transformed into natural logarithm; LF% =Low-Frequency band in normalized units; log-HF = High-Frequency band transformed into natural logarithm; HF% = How-Frequency band in normalized units; logLF/HF ratio = LF/HF ratio transformed into natural logarithm

As suggested by Laborde and colleagues [40], changes in LF% and HF% were operationalized using the delta calculation. Once the relative reactivity and recovery values ​​were obtained, a comparison between groups was carried out (using a T-Test for Independent Samples) for each PSP recording phase. Specifically, significant differences emerged considering the reactivity to Subjective Stress (Reactivity 3) and subsequent recovery (Recovery 3) (Table 4).

**Table 4:** Comparisons of delta (Δ) HRV values between the control and migraine group

	Control group (n = 30)		Migraine group (n = 30)		t (59)	*p*	Cohen’s D
	M	SD	M	SD			
LF%							
Δ Reactivity 1	-0.09	25.68	5.60	16.30	1.02	0.16	0.26
Δ Recovery 1	-4.53	26.08	-3.40	19.65	-0.02	0.49	-0.01
Δ Reactivity 2	0.73	23.72	6.31	22.22	0.97	0.17	0.25
Δ Recovery 2	-5.48	26.05	-6.79	17.24	-0.40	0.35	-0.01
Δ Reactivity 3	-10.76	21.08	2.96	21.30	1.41	0.01	0.62
Δ Recovery 3	-1.32	22.40	-10.76	21.08	-1.67	0.05	-0.43
HF%							
Δ Reactivity 1	-1.19	20.96	-7.47	17.15	-1.27	0.10	-0.33
Δ Recovery 1	4.37	21.84	6.06	17.32	0.33	0.37	0.09
Δ Reactivity 2	-4.12	21.96	-5.06	23.77	-1.16	0.44	-0.04
Δ Recovery 2	8.47	22.83	0.84	22.85	-1.29	0.11	-0.33
Δ Reactivity 3	6.99	19.99	1.01	21.72	1.11	0.14	0.29
Δ Recovery 3	-1.34	15.72	-3.66	20.03	-0.50	0.31	-0.13

## Discussion

The present study aimed to investigate HRV in different phases of a psychophysiological stress profile in a group of subjects with migraine. Therefore, the sample of migraine patients was compared with a control group, as no significant differences emerged between the two groups when looking at the socio-demographic variables, except for gender which was consistent with the literature [62]. Specifically, the objective that guided the study was to analyze the differences in reactivity to various types of induced stressors within an experimental protocol and the consequent recovery in a resting condition. Our findings highlighted that, in addition to a difference at baseline, the stressor that differentiated migraineurs from controls was the subjective one. Recounting a significant life event resulted in increased sympathetic activity in subjects with migraine compared to controls, as a significant augment in delta LF% was observed in the Subjective stressor phase.

Recent research attested that the HRV parameter is useful for describing the autonomic imbalance associated with stress response [30–32]. Specifically, the research highlighted that a high HRV is an indicator of psychophysical health, as it allows the activation of the PNS and SNS systems in a balanced manner, allowing reactivity to stressors that altered homeostasis but, also, self-regulatory and adaptability after it [63, 64]. For this reason, an optimal level of HRV is associated with self-regulation and resilience capabilities [35, 44, 45].

Looking at the migraine literature, interesting results were described by Gass and Glaross [65], who found increased sympathetic activation and reduced parasympathetic activation in a group of patients with mixed tension-type migraine by analyzing HRV. Similar findings were also collected by Perciaccante et al. [66], performing a 24-hour electrocardiographic recording. Consistent with these studies, Druschky and colleagues [67] found a higher LF and a reduced HF during a slow breathing condition, confirming a reduced parasympathetic activity in two types of neurological diseases (migraine and epilepsy), hypothesizing that psychiatric and stress comorbidities could be a common factor for both conditions.

Taking into account studies investigating the HRV parameter on headaches, the results shown by the various research groups are conflicting. For instance, a decrease in both sympathetic and parasympathetic modulation, as well as vagal tone, was described [48]. In particular, Rossi and colleagues [49] observed an alteration in autonomic modulation, with minor oscillations in both LF (sympathetic/vagal modulation) and HF (vagal modulation), consistent with previous work by Tabata and collaborators [68]. Chuang et al. [69], moreover, validated that a reduced parasympathetic/cardiovagal modulation interfered with the therapeutic response to flunarizine. In contrast to these findings, our data did not document alterations in vagal tone (HF oscillations), being more in line with Miglis et al. [70].

Our study did not detect significant differences in HRV between the control and migraine groups considering the reactivity and recovery to induced stress, except for the phase of presentation of subjective stress consisting of having to recount a personal life event. In other words, an increase in the delta LF% value was noted during subjective stress in migraine subjects, while there was a decrease in these values in control subjects. The cause of the difference between our data and those present in the literature could lie precisely in the differences between the stimuli presented, as well as the detection methods. In a similar case, Tabata and colleagues [68] suggested that fluctuations in circadian HR rhythm could be attributable to dysfunction of the serotonergic system. Thus, it is essential to bear in mind that migraine is a cyclical disorder, with changes in cortical excitability between different phases that could influence the response to stressful stimuli [71]. As an illustration, a study analyzing sympathetic and parasympathetic modulation in patients with episodic migration certified reduced autonomic activity even during the ictal phase [50].

To our knowledge, this is the first time that HRV values ​​have been compared between migraineurs and control subjects at different stages of a psychophysiological recording. Furthermore, having administered different types of stressors allowed us to highlight a different response when recounting a personal event. Specifically, while migraine sufferers perceive it as stressful to report a significant life episode, the opposite is true for healthy subjects.

As a result, this evidence enriched a line of studies that took into account the response to psychophysical stress as salient in the genesis of migraine attacks.

ANS dysfunction was repeatedly confirmed in primary headache disorders, including migraine, and is believed to be a notable aspect in headache attacks [16, 24, 28, 29]. Several studies validated a pathophysiology common to the incidence of migraine and mental suffering [72]. It seems that emotional arousal can modulate the transmission of pain, amplifying its perception [73]. First of all, it seems that a fundamental role may be played by the monoaminergic transmission that controls the descending modulation of pain, as the pathway that projects from the periaqueductal gray matter to the serotonergic neurons in the medulla and to the noradrenergic neurons in the pons and tegmentum could be altered [74]. Secondly, serotonergic transmission may be further implicated due to repeated migraine attacks causing its decrease [75]. Bearing in mind that low levels of serotonin can lead to a lowering of the pain perception threshold, the vicious circle created by depression appears evident [76]. Central monoaminergic alterations also include an imbalance between dopamine and norepinephrine in pain matrix neurons due to aberrant tyrosine metabolism, which is, in turn, consistent with dysfunctions of the HPA axis [77–79] frequently found in stress-related physical and emotional disorders [37].

In light of these assumptions, it is plausible to argue that stress is greater among migraine patients in comparison with healthy subjects as well as various psychiatric comorbidities [21, 22]. Furthermore, it is believed to contribute to the incidence, frequency, and severity of migraine attacks [15], confirming its primary role in the activation and perpetuation of attacks [24, 25].

Although our data allowed the underlining of clinically relevant findings, they must be read in light of the main limitation that characterizes HRV. It is crucial to contemplate that the HRV is a simplification of a complex bioengineering exercise and that it could be easy to confuse the part (i.e., the variable derived from the HRV) with the whole (i.e., the sympathetic/parasympathetic command) without an attitude characterized by caution. The interpretation of the LF and HF bands has not yet brought the groups of researchers to agree on the topic. By way of illustration, there is evidence that the LF oscillation corresponds to vasomotor activity and the HF oscillation corresponds to respiratory activity, casting doubt on the initial approach of splitting the frequency range [43]. More specifically, the interpretation of the LF component is the most controversial. Pomeranz et al. [79] suspected that the LF component was influenced by body position (i.e., supine or upright) while studies conducted by Malliani and colleagues as early as 1991 reassured that LF is a main indicator of sympathetic activity in healthy individuals [80], modifiable by inclination and moderate physical exercise as well as mental stress [81]. However, electrophysiological studies have also shown that LF and HF oscillations are present in both impulse activity variability signals recorded simultaneously from sympathetic and vagal efferent fibers participating in cardiac innervation, not allowing limiting LF and HF to a particular neural circuit.

Future studies could also take into account some methodological challenges that characterize PPG-based studies [52]. One limitation of PPG is that it records a delayed cardiac response farther from the heart. Thus, ECG-based estimates that present a sharp peak for the R component are not exactly matched by PPG-based methods that show a less pronounced curved peak of the signal [30]. Furthermore, ECG-based estimates of HR and HRV are recommended to obtain more reliable results because they allow visual inspection and correction of cardiac data artifacts, frequently produced in experimental protocols involving the use of stressful stimuli [56].

Lastly, the cross-sectional study design confuses the nature of the associations, which remains to be determined. Despite several studies corroborating the relationships between psychophysiological factors and headaches, it is possible to hypothesize that stress may worsen headaches which, in turn, may exacerbate emotional problems [74]. Furthermore, the small sample size and imbalance in favor of women represent a further limitation that future studies should overcome.

Nevertheless, the possible clinical implications arising from our findings could be considerable. Highlighting the need to evaluate the role of specific psychological factors and stress on the ANS could explain the manifestations of headaches and help identify patients who are more difficult to treat and who require psychological as well as medical intervention.

## Conclusions

Our study aimed to compare HRV in different phases of a psychophysiological evaluation with stressor and rest conditions in a group of migraineurs and a group of control subjects. We found that migraineurs have a different autonomic regulation with higher reactivity in the condition of a subjective stressor. In other words, recounting a significant life event is considered as stressful as a cognitive task. During an experiment where migraineurs were asked to talk about a personal episode, higher emotional involvement was observed. Autonomic imbalance may indicate greater vulnerability to stress and, therefore, lower adaptability and resilience. Our findings are in line with the research on the psychophysiology of migraine, which supports that stress and autonomic imbalance can influence the incidence, frequency, and intensity of migraine attacks.

## Electronic supplementary material

Below is the link to the electronic supplementary material.

Supplementary Material 1
